# “MetasTAZation” and beyond

**DOI:** 10.18632/oncoscience.185

**Published:** 2015-08-11

**Authors:** Monica Bartucci

**Affiliations:** Rutgers Cancer Institute of New Jersey, New Brunswick, NJ, USA

**Keywords:** TAZ, metastasis, breast cancer

In the last decade, many research studies suggested that solid tumors are hierarchically organized and sustained by a distinct pool of quiescent, therapy-resistant and highly aggressive cells identified as cancer stem cells (CSCs).

In addition to being responsible for tumor initiation and growth, CSCs are expected to mediate cancer spread and relapse. While the scientific community is still debating on the legitimacy of the CSCs theory, an increasing amount of evidence indicates that their existence appears certainly valid in some cases. For instance, in breast cancer (BC) the relapse rate following therapy regimens can exceed 50% and may occur even 30 years after the initial diagnosis. Thus, one could speculate that conventional treatments might spare a small population of therapy-resistant cells that “quiescently” await until the right moment to re-initiate tumor growth; with these cells be, by definition, CSCs.

Again in mammary tumors, metastatic growth has been proposed for many years as an exclusive property of CSCs, venturing that their biological plasticity may ease survival in secondary organs. Nevertheless, the proof of their identity as cells of origin of recurrences at distant sites was only recently given when, by employing fluorescent-labeled patient-derived cells and developing an *in vivo* metastatic model simulating BC clinical course, we show that, unlike the rest of the tumor bulk, only CSCs are capable to colonize distant organs [1].

Metastasis is an extraordinarily complex process responsible for nearly 90% of cancer-related deaths. High throughput technologies widened our comprehension on the genetic and the biochemical determinants associated with cancer development and progression [2]. Yet, the relevance of single pathways on metastatization is still unclear, especially when pondered within the pyramidal organization of tumors. Among others, the Hippo signaling is emerging as a fascinating oncogenic route mediating a variety of tumor-promoting functions such as epithelial-to-mesenchymal transition (EMT), therapeutic resistance and, accordingly, CSC generation [3, 4]. Thus, it was not totally surprising when a gene-expression profile comparison between metastagenic and non-metastagenic cells in BC linked one of the major Hippo signaling components, TAZ, to the metastatic ability of breast CSCs, along with their chemoresistance and tumorigenic potential.

Genetic downregulation of TAZ expression level sensitized CSCs and relative tumor xenografts to chemotherapy, suggesting that the presence of this protein in CSCs contributes considerably to drug resistance in BC. More importantly, it led to the observation that metastases appearance was remarkably repressed in TAZ depleted CSCs, regardless the number of cells injected. These data were particularly significant because, although previous work suggested a relationship between TAZ expression and poor patient outcome in BC [5], a functional role of TAZ in tumor metastasis was not previously reported.

TAZ is tightly linked to EMT, a complex cellular program well known for its association to epithelial plasticity and BC stemness [5, 6]. Accordingly, we observed that upon TAZ overexpression, non-tumorigenic BC cells developed a more spindle-like shape *in vitro,* suggesting an EMT transition, but also acquired tumorigenic capacity *in vivo*. To this regard, our and previous studies suggest a scenario in which, by inducing morphological changes, EMT determine TAZ stabilization and, in turn, acquisition of CSC, drug resistance and metastatic properties [1, 5].

Collectively, these data indicate that TAZ is instrumental in inciting metastasis in gain- and loss-of-function assays in mouse models, but does it correlate with overt metastatic lesions in human patients?

The understanding of the biology of TAZ and the molecular outputs it elicited in our preclinical model, has encouraged a series of clinically focused analyses aimed at explore the prognostic/predictive significance of this protein. Initially we selected a number of primary BCs along with their paired metachronous metastases and observed that TAZ is overexpressed in breast secondary lesions. Then, to assess whether our findings had clinical relevance, we determined TAZ expression in a larger cohort of BCs patients with complete follow up data. We observed that high TAZ levels represent an independent negative prognostic factor together with the presence of metastatic lymph nodes and negativity for estrogen receptors [1].

Additionally, since some of our unpublished results revealed significant correlation between TAZ and HER2 positivity, in a follow up study we also investigated the association between TAZ expression and the pathological complete response in a subset of HER2-positive BCs. Results collected in this second study also suggest that the TAZ score efficiently predicts pathological complete response in Luminal B, HER2-positive BC patients previously treated with chemo- and targeted-therapy [7], designating a novel role for this protein in the clinical diagnostic.

In conclusion, the understanding of the biological relevance of the Hippo pathway in cancer is very recent. The data collected so far from both preclinical and clinical studies, indicate that it deserves further and more thorough investigations, especially in BC, where TAZ holds the potential to implement the current pipeline of biomarkers and, possibly, therapeutic targets.
